# MoLPC2: improved prediction of large protein complex structures and stoichiometry using Monte Carlo Tree Search and AlphaFold2

**DOI:** 10.1093/bioinformatics/btae329

**Published:** 2024-05-23

**Authors:** Ho Yeung Chim, Arne Elofsson

**Affiliations:** Science for Life Laboratory and Department of Biochemistry and Biophysics, Stockholm University, Stockholm 106 91, Sweden; Science for Life Laboratory and Department of Biochemistry and Biophysics, Stockholm University, Stockholm 106 91, Sweden

## Abstract

**Motivation:**

Today, the prediction of structures of large protein complexes solely from their sequence information requires prior knowledge of the stoichiometry of the complex. To address this challenge, we have enhanced the Monte Carlo Tree Search algorithms in MoLPC to enable the assembly of protein complexes while simultaneously predicting their stoichiometry.

**Results:**

In MoLPC2, we have improved the predictions by allowing sampling alternative AlphaFold predictions. Using MoLPC2, we accurately predicted the structures of 50 out of 175 nonredundant protein complexes (TM-score ≥ 0.8) without knowing the stoichiometry. MoLPC2 provides new opportunities for predicting protein complex structures without stoichiometry information.

**Availability and implementation:**

MoLPC2 is freely available at https://github.com/hychim/molpc2. A notebook is also available from the repository for easy use.

## 1 Introduction

Proteins serve critical functions in various biological processes, including mRNA splicing (Will and Lührmann 2011), protein degradation (Tanaka 2009), and assisting protein folding (Ditzel *et al.* 1998). However, predicting a protein's 3D structure from its amino acid sequence remained challenging until the release of AlphaFold (Jumper *et al.* 2021, Evans *et al.* 2022). Proteins often form complexes with specific biological activities to perform their molecular functions in the cell (Goodsell and Olson 2000). Although AlphaFold can often predict the structure of single and multiple-chain proteins with remarkable accuracy (Akdel *et al.* 2022, Zhu *et al.* 2023), it is limited to predicting small complexes that fit into the memory of the GPU. Further, modelling methods cannot predict the structure of a protein complex when the stoichiometry is unknown (Bryant *et al.* 2022b, Evans *et al.* 2022, Li *et al.* 2022, Pao-Huang *et al.* 2023, Shor and Schneidman-Duhovny 2024).

This paper proposes an enhancement to MoLPC (Bryant *et al.* 2022b), named MoLPC2, which can model large protein complexes without stoichiometry information. To the extent of our knowledge, MoLPC2 is the first protein complex prediction method that does not depend on any symmetry or stoichiometry information. MoLPC2 also enhances the functionality of MoLPC by using structural analysis of the sub-component, resulting in an increased speed and better performance. We have evaluated the performance of MoLPC, MoLPC2, and AlphaFold-Multimer by assembling 175 nonredundant protein complexes and aligning the predictions to the corresponding native structures in the Protein Data Bank.

## 2 Materials and methods

### 2.1 Benchmarking

This study uses the same large protein complex dataset previously used to benchmark MoLPC. This dataset comprises 175 nonredundant protein complexes with 10–30 chains obtained from the Protein Data Bank (PDB). Most of these protein complexes contain 10–12 chains and exhibit dihedral and cyclic symmetry. The global symmetry definitions were derived from the PDB annotation, and the structures were obtained from the first biological assembly. For more information on replicating this dataset, please refer to the MoLPC paper (Bryant *et al.* 2022b). MMalign (Mukherjee and Zhang 2009) is used to evaluate the prediction computing the MMscore normalized to be between zero and one, where one indicates a perfect match to the structure from PDB.

### 2.2 MoLPC2

MoLPC2 consists of six calculation steps, outlined below (Supplementary Material and Supplementary Fig. S1).

(i) The prediction of stoichiometry is performed by first computing all possible combinations of sub-components. (ii) Next, these sub-component structures are predicted using AlphaFold-Multimer (AFM). (iii) Sub-component duplicates are filtered out using a structural similarity filter. (iv) Interaction pairs are generated from the sub-components, and pairs with a minimum difference are removed using a filtering step. (v) The remaining pairs are used to assemble the protein complex using the Monte Carlo Tree Search (MCTS) algorithm. The MCTS will iteratively simulate the random addition of new chains and update the search tree to find the optimal decision. (Supplementary Material and Supplementary Fig. S2). (vi) Finally, the protein complex is scored using the pDockQ score (Equation 1). In contrast to MoLPC, the same pair can be used multiple times, enabling the prediction of stoichiometry. Adding new chains is continued until no more chains can be added or the maximum number of chains is reached.

(1) log 10(no. of interface contacts)×avg interface plDDT

## 3 Results and discussion

The prediction accuracy of MoLPC2 is improved compared to MoLPC. There are mainly two contributors to the improved performance: assembling from larger sub-components and sampling more predictions from AlphaFold-Multimer.

MoLPC2 has enabled the assembly of protein complexes from larger sub-component structures (4mers), previously hindered by the limitations in protein prediction size using FoldDock (Bryant *et al.* 2022a). As shown in the MolPC paper (for 3mers over 2mers), utilizing larger sub-components allows for additional interactions, some of which may not be predicted from smaller sub-component sizes. Further, by employing tetramer instead of trimer sub-components, the mean TM-score of MoLPC2 improved marginally from 0.49 to 0.54 (Table 1).

**Table 1.** Benchmark results of the different complex modelling methods.^a^

Methods	Average <MMscore>	Correct stoichiometry	Success rate (%)
MoLPC^b^	0.47	N/A	20
AFMv2.3^b^	0.69	N/A	40
CombFold^b^	0.69	N/A	43
Score-based diffusion model^b^	0.32	N/A	<5
MoLPC2 with stoichiometry^b^	0.58	N/A	32
MoLPC2 (3mer)	0.48	24	17
MoLPC2 (3mer + 5 models)	0.49	19	16
MoLPC2 (4mer)	0.53	29	23
MoLCP2 (4mer + 5 models)	0.57	39	30

a Comparisons between different complex structures prediction methods. Predictions with MMscore > 0.8 are assumed to be successful.

b Indicates that the stoichiometry is assumed to be known.

In MoLPC2, an improved approach is used to extract interaction information from sub-component structures. Unlike MoLPC, which employs only one prediction for each sub-component in assembling, MoLPC2 utilizes all five predictions from the five different AlphaFold-Multimer (AFM) models, as the predictions from different AFM models may differ for the same sub-component, and the ranking by AFM is not always optimal for selecting a sub-component for assembling. Recent studies have shown that utilizing all predictions, regardless of their ranking, can improve accuracy for multimer predictions (Wallner 2023). By using all five predictions for sub-component prediction, MoLPC2 can rescue some protein complexes that could not be assembled using only the best-ranked prediction from AFM (Supplementary Fig. S3).

However, using larger sub-components and including all sub-component predictions for assembling in MoLPC2 may lead to longer search times. Therefore, to improve MCTS efficiency, a strategy is employed to select chains with some interface contacts less than or equal to the minimum interface contacts plus one for adding a new chain (Supplementary Material and Supplementary Fig. S4). This approach reduces the number of chains searched for addition. However, this strategy is only applicable to symmetrical proteins. For asymmetrical proteins, this strategy may not aid MCTS in the search for the appropriate move, as the chains in asymmetrical proteins usually lack a balanced number of interface contacts. After implementing above features, the structural prediction was performed on one NVIDIA A100 GPU with 40 Gb of RAM with a time limit of 72 h per prediction (Supplementary Material).

Even without the stoichiometry information, MoLPC2 can still obtain on-par performance compared to MoLPC for most of the targets. However, in the case of stoichiometry information, AFM still outperforms both versions of MoLPC, but it still has a size limit not present in MoLPC (Supplementary Fig. S5).

### 3.1 Symmetry

**Figure 1.**
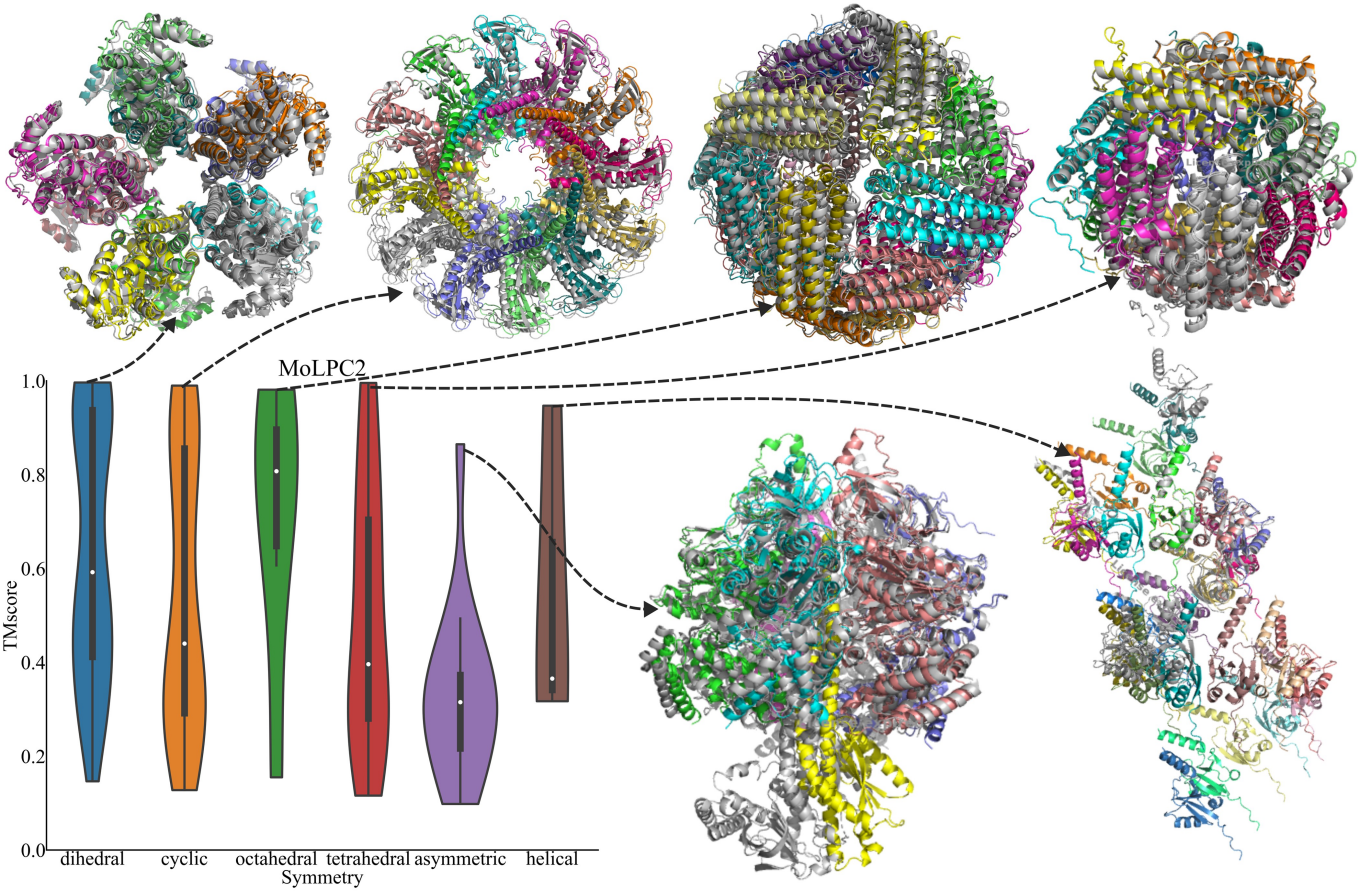
MoLPC2 predicting protein complexes with different symmetry classes. Distribution of TM-scores and examples of the best assemblies for each symmetry type. The assemblies are coloured by chain, and the true complexes are in structural superposition in grey. The structures shown for each symmetry and the corresponding TM-scores are: 5BSE (Dihedral, 0.99), 1IJG (Cyclic, 0.99), 1MFR (Octahedral, 0.98), 1DPS (Tetrahedral, 0.95), 5XPB (Helical, 0.95), 2V7Q (Asymmetrical, 0.86).

Applying MolPC2 to large complexes with different symmetries shows that the methodology works best for symmetric complexes. Remarkably, within each symmetry class in the dataset, at least one protein complex has been successfully predicted by MoLPC2. This observation suggests that the symmetry classification of the protein complex does not constrain the predictive capabilities of MoLPC2. Nevertheless, accurately predicting asymmetric structures remains a formidable task for MoLPC2 due to inherent challenges such as unknown stoichiometry and the exponential increase in computational time resulting from a substantial number of distinct chains (Fig. 1).

## 4 Conclusions

It is challenging to predict the structure of large protein complexes without prior knowledge of their stoichiometry. This study presents an improved version of the Modelling of the Large Protein Complex (MoLPC) method, named MoLPC2. This method utilizes Monte Carlo Tree Search and AlphaFold to assemble protein complexes from sub-components and simultaneously predict their stoichiometry.

Our results demonstrate that MoLPC2 performs well in predicting symmetrical protein complexes without knowing their stoichiometry. However, accurate prediction of protein complex structures with MoLPC2 relies heavily on the performance of AlphaFold-Multimer. We observe a clear correlation between the performance of these two methods, particularly in predicting asymmetrical protein complexes (Supplementary Figs S6 and S7).

Although MoLPC2 may not always predict the complete structure of a protein complex, it provides valuable insight into the correct stoichiometry. Monte Carlo Tree Search employed by MoLPC2 can predict a portion of the final protein complex in cases where the method performs modestly. The stoichiometry can be estimated from the incomplete structure, which can then be used with AlphaFold-Multimer to predict the complete structure (Supplementary Fig. S8).

Nevertheless, a constraint of MoLPC2 lies in its prerequisite of prior knowledge of the protein complex. It necessitates knowing which protein sequences will likely form a protein complex to utilize MoLPC2 efficiently. This constraint arises due to the many possible combinations, which can lead to computationally expensive predictions. Although some methods aid in predicting interacting protein pairs, searching for interacting protein pairs from a vast pool of proteins remains challenging. If this limitation can be overcome, it would enable the complete proteome, including protein complexes, to be constructed solely from the genome.

## Supplementary Material

btae329_Supplementary_Data

## Data Availability

All data and code used in this study is available from https://github.com/hychim/molpc2/.
