# Supplementary material for: MoLPC2: improved prediction of large protein complex structures and stoichiometry using Monte Carlo Tree Search and AlphaFold2
Source: Bioinformatics. 2024 May 23;40(6):btae329. doi: 10.1093/bioinformatics/btae329 (PMC11194477; doi:10.1093/bioinformatics/btae329)
Supplement: btae329_Supplementary_Data [file btae329_supplementary_data.pdf]

1. Supplementary Figures

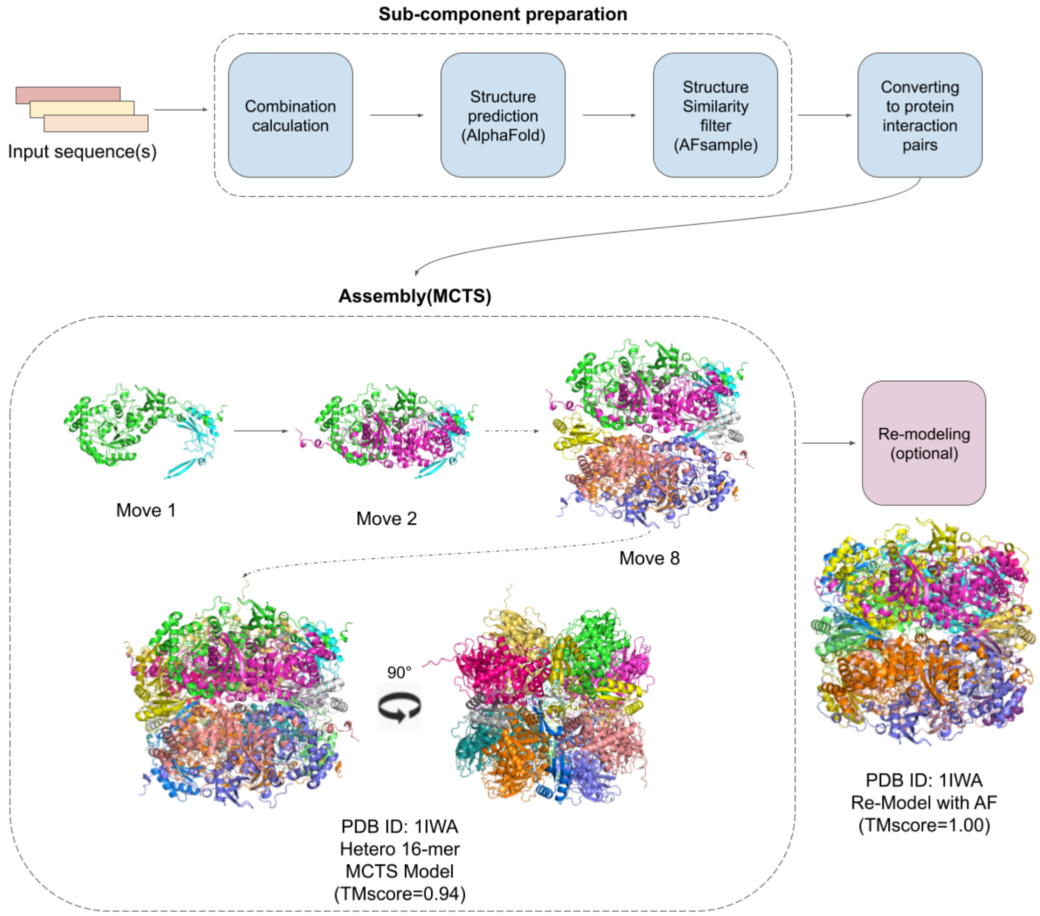

Supplementary Figure 1: MoLPC2 pipeline overview. The sequences of individual chains will be first converted to combinations of sub-components that can be formed. After that, the sub-component sequence combinations will be used for predicting the structure of the sub-component in AlphaFold-Multimer. 5 models from AlphaFold-Multimer will be utilised, and all predictions will be passed through a structural similarity filter to remove structures that have high similarity. After structures with low similarity are filtered out, all sub-components are converted into protein-interacting pairs. In contrast, pairs with distances larger than 8A will be classified as non-interacting pairs and filtered out. All remaining interacting pairs are then used as possible moves for assembly in MCTS. After several repeats of MCTS, the structures are assembled and re-model with IMP or AFM. The example structure shown in the MCTS is Ribulose-1,5-bisphosphate carboxylase/oxygenase (PDB code: 1IWA), which is a hetero 16-mer and has dihedral symmetry.

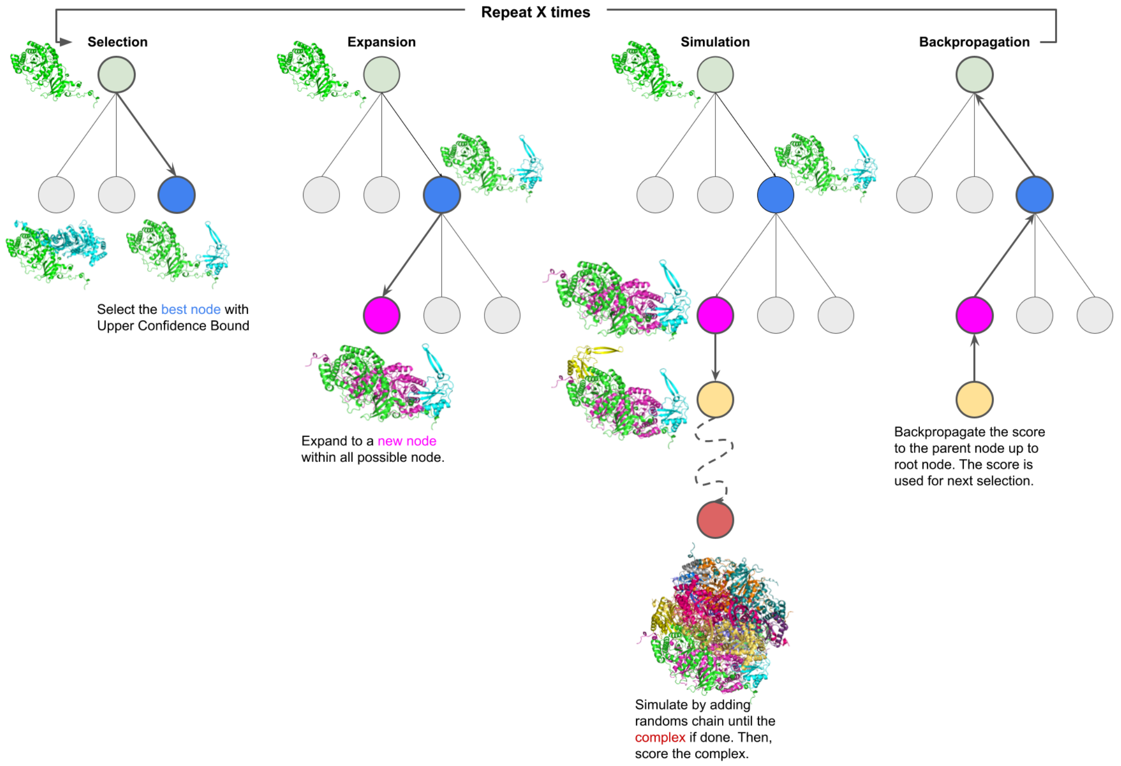

Supplementary Figure 2: Monte Carlo Tree Search algorithms in MoLPC2. Starting from a node (sub-complex), a new node is selected based on the previously back-propagated scores. From this node, a random node is added (expansion). A complete assembly process is then simulated by adding nodes randomly until an entire complex is assembled or a stop caused by too much overlap is reached. The complex is scored, and the score is back-propagated to all previous parent nodes, which yields support for the following selections. The final result is that the nodes most likely to result in high-scoring complexes are joined in a path containing all chains. The principle for the complex 1IWA is shown.

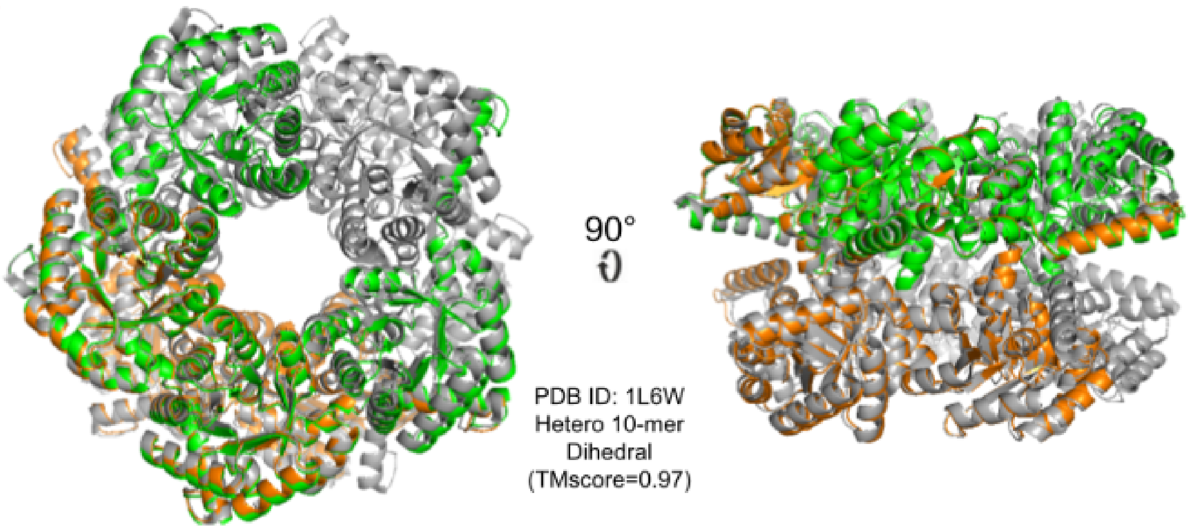

Supplementary Figure 3: Aggressive sampling from AFM predictions. In MoLPC2, an improved approach is used to extract interaction information from sub-component structures. Unlike MoLPC, which employs only one prediction for each sub-component in assembling, MoLPC2 utilises all five predictions from the five different AlphaFold-Multimer (AFM) models. This is because the predictions from different models in AFM may differ for the same sub-component, and the ranking by AFM is not always the best for selecting a sub-component for assembling. By using all five predictions for sub-component prediction, MoLPC2 can rescue some protein complexes that could not be assembled using only the best-ranked prediction from AFM. For example, the 10-mer protein complex 1L6W is shown here. The best-ranked prediction of the 0-0-0-0 sub-component resulted in incomplete assembly (coloured in green), with only one layer of the dihedral structure assembled. By incorporating different predictions from AFM (coloured in orange), a wider variety of interaction choices is achieved, and the assembly of protein structures is better, as exemplified here. The native structure is coloured in grey.

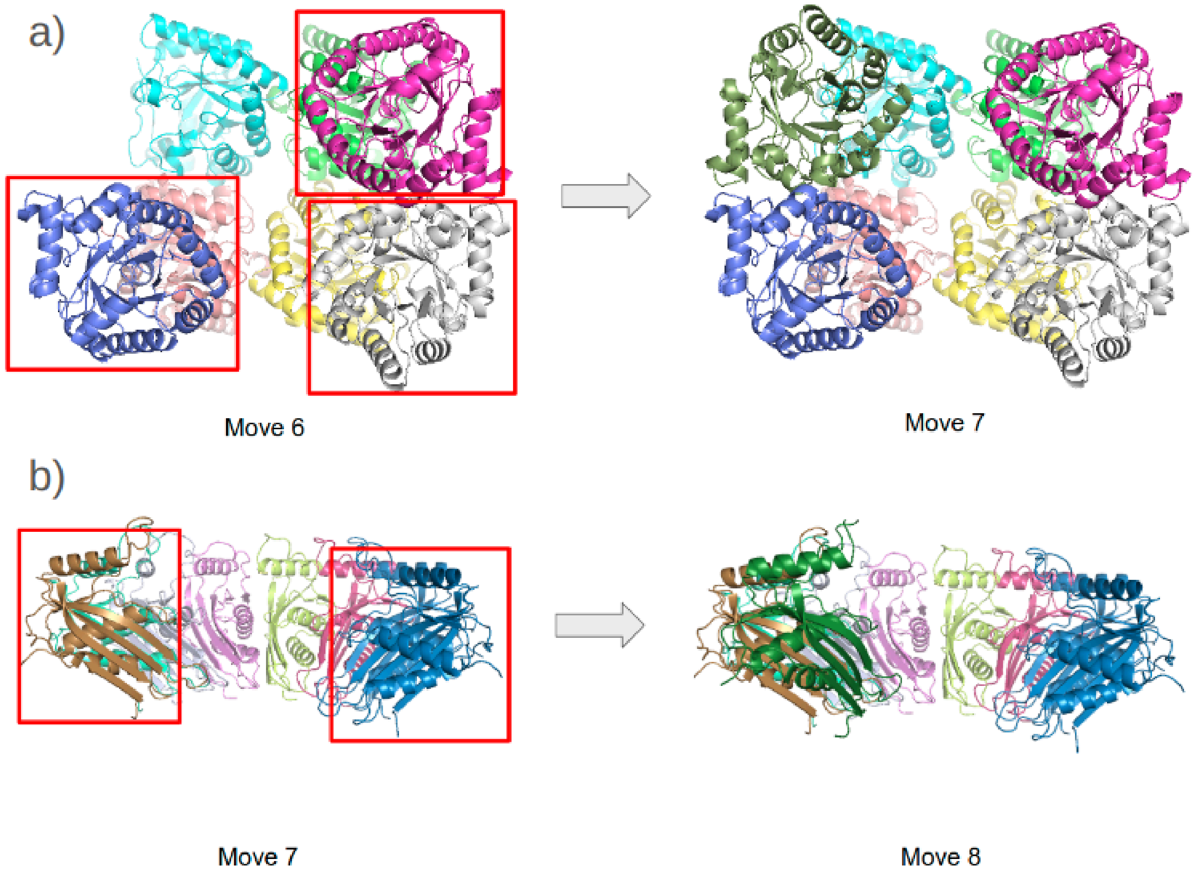

Supplementary Figure 4: Structural analysis for searching filtering in MTCS. To improve the efficiency of the MCTS, an approach to reducing the number of unlikely moves has been taken. Only chains with [minimum interaction +1] will be selected for adding new chains. The idea is inspired by how the human brain would add chains to a complex. For example, for a halfway-finished protein, it is easy for humans to think we should add chains on the two ends to assemble the structure. A) An example of a dihedral protein, 1M3U, is shown. Only chains within the red box will be searched for the addition of new chains. The chains in the middle of the structure are ignored because of their high number of interactions and are assumed to be less likely to have another chain interacting with them. b) An example of cyclic protein, 1QAW, is shown. Only chains in the red box will be searched for the addition of new chains because of the same reason mentioned.

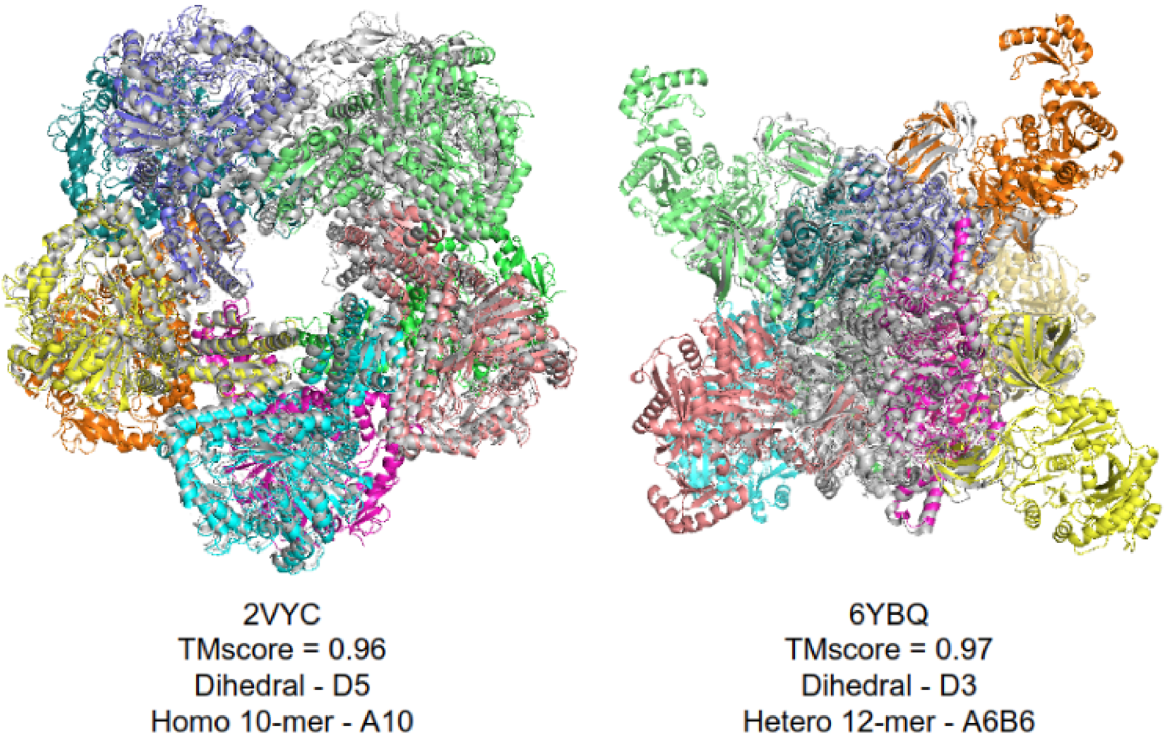

Supplementary Figure 5: Very large protein complex. The latest version of AlphaFold-Multimer (AFM) has significantly increased the upper limit on the number of protein residues that can be predicted. However, very large protein complexes still exist that cannot be predicted with AFM due to their size or the long computational time required. In contrast, MoLPC2 can predict such large protein complexes by assembling them from sub-components, which generally have smaller sizes than the whole complex. Two examples are shown here where AFM fails to make predictions due to exceeding the maximum residues limit or lengthy computational time but can be predicted using MoLPC2. The predicted structure on the left is 2VYC and failed to be modelled using AFM because of exceeding GPU memory in NVIDIA A100 Tensor Core GPUs with 40GB memory. The predicted structure on the right is 6YBQ and failed to be modelled using AFM because of long computational time and time out. Both of their structures are predicted successfully using MoLPC2. Each chain is coloured differently, while the native structure is grey.

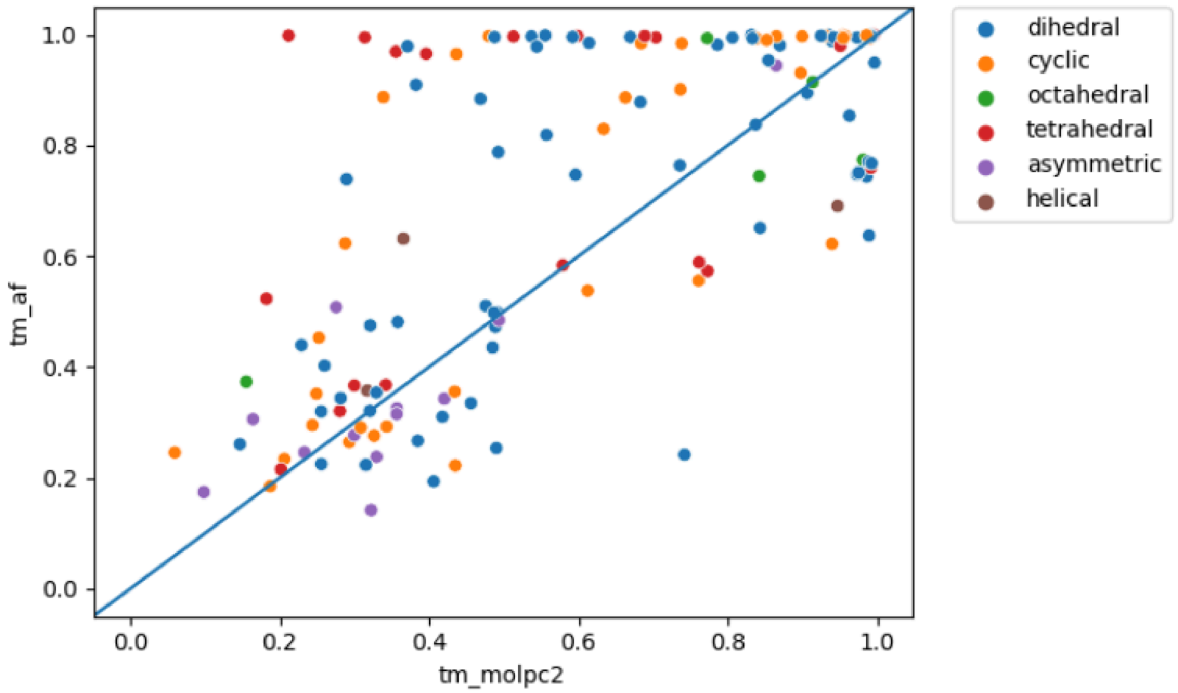

Supplementary Figure 6: TMscore of AFM predictions vs TMscore of MoLPC2 predictions. The sample is coloured by symmetry class. The line shows a linear relationship. AlphaFold-Multimer performs well in predicting the structures of protein complexes with symmetrical symmetry. However, its accuracy in predicting the structures of asymmetric protein complexes is comparatively lower than that for other types of protein complex symmetry. This performance discrepancy may arise because asymmetric protein complexes exhibit unique behaviours distinct from symmetrical protein complexes, making it more challenging for AlphaFold to learn and search for multiple sequence alignment (MSA) information. Nevertheless, many asymmetric protein complexes still possess symmetric regions, and AlphaFold can predict these regions with near-perfect accuracy. In the majority of cases, AlphaFold-Multimer exhibits superior performance compared to MoLPC2. However, it should be noted that AlphaFold- Multimer necessitates protein stoichiometry, whereas MoLPC2 does not. It is also evident that when AlphaFold-Multimer produces inaccurate predictions, MoLPC2 demonstrates sub-optimal performance, given its strong dependence on the precision of sub-components predicted by AlphaFold-Multimer.

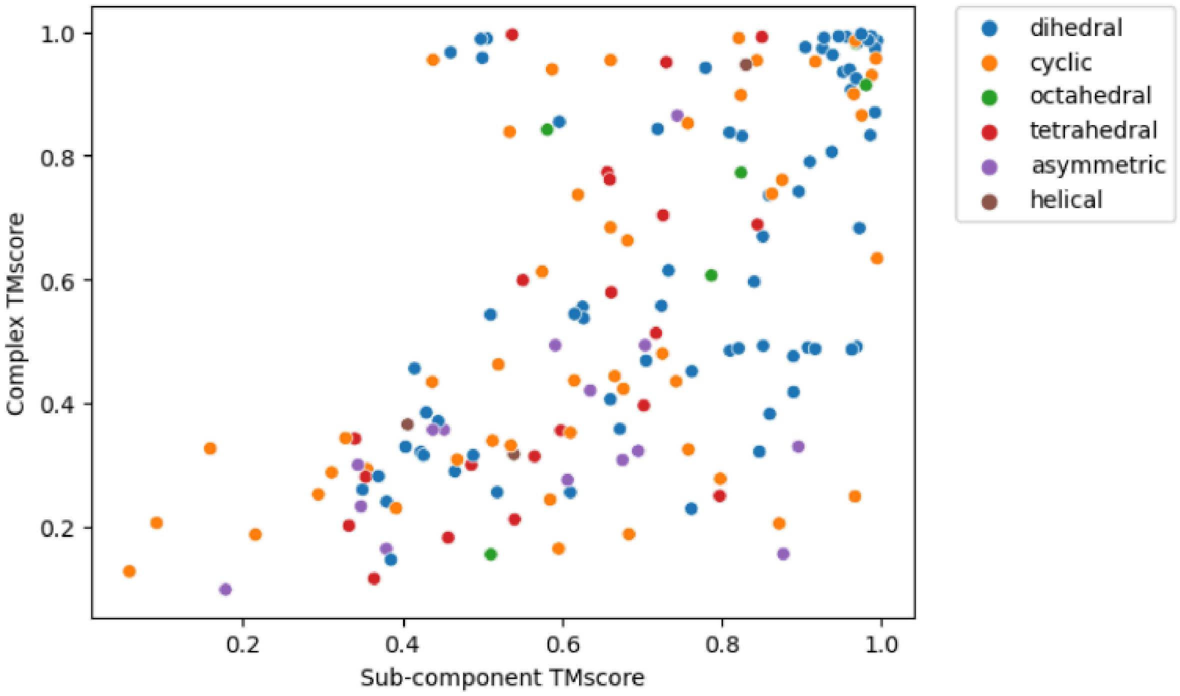

Supplementary Figure 7: Average TM-score of sub-components vs TM-score of final prediction complex. The sample is coloured by symmetry class. Pearson correlation = 0.6088. The average TM-score of the sub-components determines the most apparent indicator of the accuracy of an assembled protein complex. In cases where the sub-components exhibit high levels of accuracy, the assembled complex is also highly accurate. However, this criterion is not universally applicable to all predictions made by MoLPC2. Although some predictions display a high average TM-score of sub-components, their overall complex TM-score remains low, indicating that they do not possess every correct interaction in the native structure and cannot be assembled correctly.

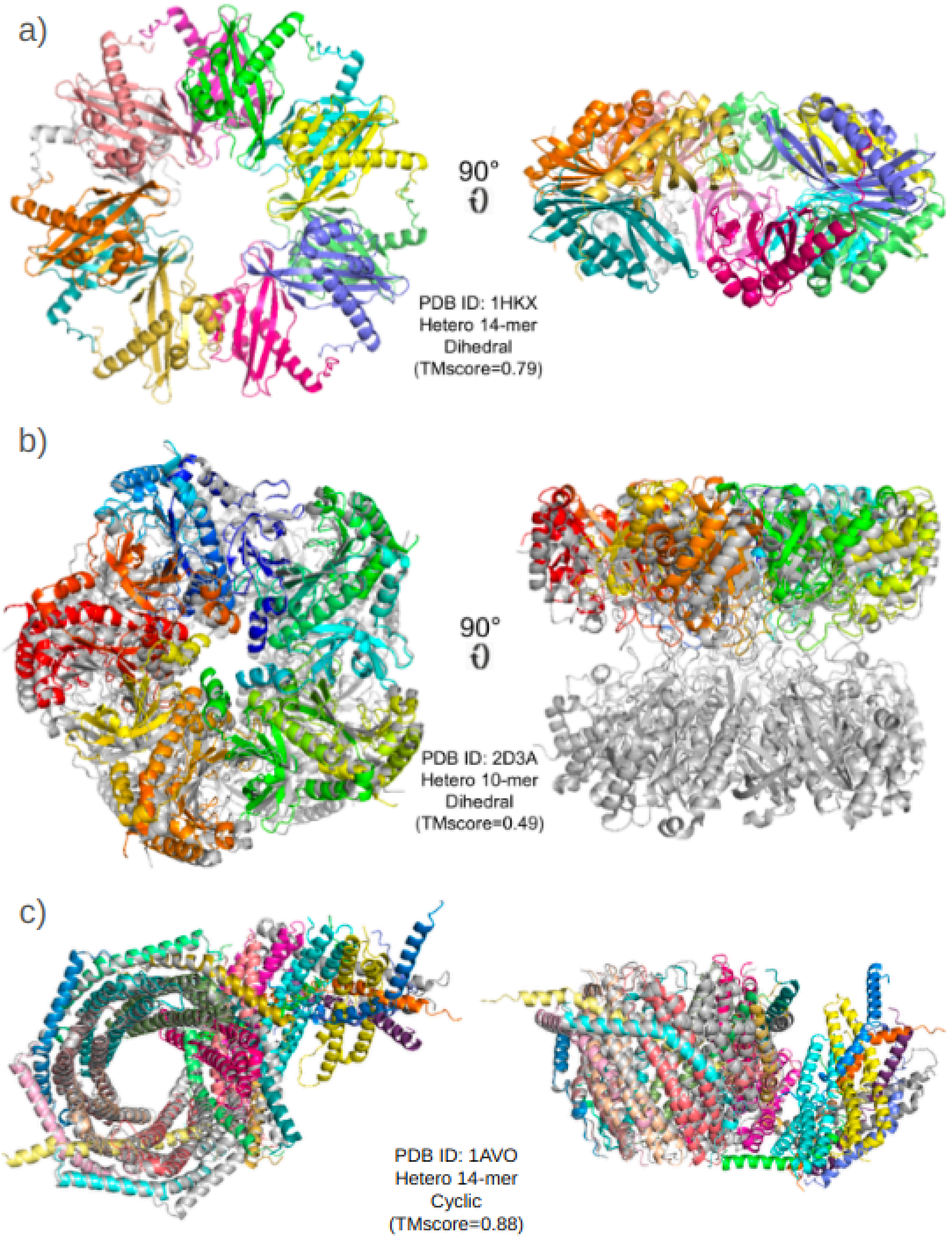

Supplementary Figure 8: Example of different error types with incorrect stoichiometry. Model coloured by the chain. Chains colour predicted models, and native structures are coloured grey. a) 1HKX model predicted using MoLPC2. Even MoLPC2 cannot assemble the entire structure, but a significant portion is still assembled, and the symmetry of the complex is correctly predicted. The protein complex will likely be dihedral with 14 chains. MoLPC2 predicted only 12 with correct D7 symmetry. b) 2D3A model predicted using MoLPC2. MoLPC2 can only assemble half of the structure. Based on prior biological knowledge of the protein function, it would only be possible to infer that the protein exists in a dihedral symmetry rather than cyclic, with twice the number of chains. c) 1AVO model predicted using MoLPC2. c) 1AVO model predicted using MoLPC2. Extra parts are assembled using MoLPC2, causing the prediction to have a larger stoichiometry than the native structure.

However, the main part of the protein is still assembled successfully with a TMscore>0.8. Remodeling with predicted stoichiometry using AFM would improve the quality of the prediction.

Supplementary information

1.Methods

1.1 Overview

We called the improved pipeline MoLPC2, which can model protein complexes without protein stoichiometry using Monte Carlo Tree Search (MCTS) and AlphaFold-Multimer. The only required input for the MoLPC2 pipeline is a fasta file with all individual chains' sequences. The sequences will then calculate all possible combinations forming a sub-component. The sub-component sequences will then be inputted to AlphaFold2 to predict the structure of the sub-component. The structure will then be filtered under a structural similarity filter to remove structures that have high similarity and keep the sub-component fold as diverse as possible. The filtered sub-component structure will then be converted into interacting pairs for assembly in MCTS. After the MCTS, we can get the assembled protein complex and the assembling path. The assembling path and extracted distance from the complex can also be used for re-modelling in AFM. Fig S1 shows the overview of the MoLPC2 pipeline.

1.2 Subcomponent combination

The ability to manually assemble large protein complexes from sub-components (Burke et al., 2023) or using Monte Carlo Tree Search has been previously demonstrated (Bryant, Pozzati, Zhu, et al., 2022). The sub-component is a critical factor in the assembly process. Since the stoichiometry of the complex is usually unknown, all possible combinations of sub-components are first calculated before the structures of sub-components are predicted in subsequent steps. For instance, for a heteromer with four unique chains, 20 and 35 sub-component structures will be required to predict sub-components with three and four chains, respectively. The number of necessary sub-components to assemble a complex with n unique chains from sub-components with k chains can be computed using the following equation:

$$\binom{n+k-1}{k} = \frac{(n-1+k)!}{(n-1)!}$$

The combination of sub-component sequences is calculated using the Python package itertools combinations with replacement function, for example, for a protein complex with two individual chains(chain 0 and chain 1). The final combination of

trimer sub-components will be 000, 001, 011 and 111. (Sub-component 000 here means a sub-component with three identical chains 0.)

The Python code used is shown below:

```
from itertools import combinations_with_replacement

mer = 3 # number of chains in the subcomponent
list = [0 , 1, 2] # list of individual chains in the complexes
comb = [p for p in combinations_with_replacement(list , mer)]
```

Sub-component modelling and protein interaction extracting.

1.2.1 AlphaFold-Multimer

Since we use a conda environment is used in this project instead of a Docker container, the official script that is utilised to run AlphaFold with Docker cannot be utilised. Another community version of the AlphaFold script (https://github.com/kalininalab/alphafold non-docker) is used instead. The community version also using a conda environment and uses bash script instead of python script to run the AlphaFold. We run the AlphaFold for every combination of sub-component fasta using this command:

```
bash run_alphafold.sh -d ../alphafold_data_v2.3 -o
output/$ID/alphafold/ -f data/$FASTA -t 2021-11-01 -r false -m
multimer -l 1
```

The sub-components are fed directly into AlphaFold-Multimer to predict their structures. In MoLPC2, AlphaFold-Multimer (version 2.3.1) is utilised for predicting sub-component structures. The latest version of AlphaFold-Multimer (version 2.3) has lifted the limitation on predicting larger sub-components, which can improve the assembly process by discovering and utilising more interactions. The default parameters for AlphaFold-Multimer are used, except for the number of predictions from models changed from five to one, and relaxation is turned off.

To ensure that MCTS assembles the structure correctly, it is essential to have all the correct interactions. Therefore, predictions from all five models are kept instead of using only the best model. This approach aids in rescuing some previously impossible proteins to model due to the best model not including all correct protein interactions. However, since predictions from different models are generally very similar, a filter is necessary to eliminate similar structures and enhance the efficiency of MCTS.

1.2.2 Sub-component structural similarity filter

In this study, an approach similar to that used in AFsample (Wallner, 2023) is employed to enhance the performance of Monte Carlo Tree Search (MCTS) by filtering out similar protein sub-components. The primary goal is to reduce the number of similar moves and protein pair choices, thus enhancing the efficiency of MCTS. Without filtering, many possible actions must be expanded first, leading to longer running times for MCTS.

The first step in the filtering process is to use MMalign (Mukherjee & Zhang, 2009) to calculate the TM-score between all sub-component structures. Those with a TM-score greater than 0.9 to another sub-component are then deleted. All remaining sub-components are assumed to have different folds, allowing the MCTS algorithm to have a broader choice of protein interacting pairs. This helps model proteins with other sub-component predictions in AlphaFold-Multimer and speeds up MCTS by removing similar interactions.

```
comb = [p for p in combinations(subcomponent), 2)]
for pairs in comb:
      tmscore = run_mmalign(pairs A , pairs B)
      if tmscore > 0.9:
           remove( pairs B )
```

1.2.2.1 Converting sub-components to interaction pairs

Following the filtering process, converting the protein sub-components into protein interaction pairs is necessary for superimposing in MCTS. The protein pairs are initially computed from the sub-component, and the minimum distance between the chains is calculated. Subsequently, pairs with a minimum distance exceeding 8 Å are eliminated, while those that remain are presumed to be interacting pairs that can be reassembled into the sub-component structure.

The protein interaction pairs are identified by their unique chain ID and PDB file chain id for differentiation in MCTS. In this study, the unique chains are designated using numerical labels (e.g. 0, 1, 2), while protein chains are designated using alphabetical order (e.g. A, B, C). For instance, a protein dimer comprising both unique chain 0 and having PDB chain id B and D will be labelled as 0-0 and B-D, respectively. This step is critical and represents a key factor in enabling our pipeline to assemble proteins without prior knowledge of stoichiometry.

1.3 Protein complexes assembly path search (MCTS)

Monte Carlo Tree Search (MCTS) is a powerful decision-making algorithm that has gained widespread recognition in artificial intelligence, particularly in game-playing agents. The algorithm is designed to find the optimal solution by exploring the search space and selecting the best possible move at each game step (Metropolis & Ulam, 1949). MCTS has been successfully applied in a variety of domains, including chess games (Silver et al., 2017), poker games (Van den Broeck et al., 2009), robotics (Labbé et al., 2020), and even medical decision-making (Sadeghnejad-Barkousaraie et al., 2020).

The core idea of MCTS is to build a tree structure that represents the possible moves and outcomes of a game. The tree is constructed by performing a series of simulations, or rollouts, starting from the root node and randomly selecting actions until a terminal state is reached. The outcomes of these simulations are then used to update the tree structure, which allows the algorithm to focus its search on the most promising areas of the search space (Metropolis & Ulam, 1949). MCTS has several advantages over traditional search algorithms, including handling large and complex search spaces, adapting to changing environments, and making decisions based on incomplete or uncertain information.

Due to the large number of possible paths to explore in protein complex assembling, searching all assembling paths is unfeasible. Therefore, we search for an optimal path using Monte Carlo Tree Search, which has been successfully applied to solve various game-related problems.

The MCTS consists of 4 steps: selection, expansion, simulation (rollout) and backpropagation. Applying MCTS on proteins would be different from other applications(e.g., games). Usually, other applications have a clear way to generate a game tree from which MCTS can search. For example, when MCTS is applied to playing chess, the move is defined as the player moves, and the number of wins defines the score. However, applying Monte Carlo Tree Search to protein assembling is not direct since there is no ”turn” and ”player”. In the following section, each step will explain in detail how MCTS is applied to protein assembly using a simplified example (Figure S2).

For the expansion step, possible moves needed to be listed for the tree-searching algorithms to start with and select. For the root node, unique chain 0 is assumed to be the first chain in the complex. Protein interaction with chain 0, as the first chain, will be selected and used for possible moves. There is only a unique chain 0 in our ’uncompleted complex’ for protein complexes that need to be added. There will be possible moves, 0-0, 0-1 0-2, etc., and for each 0-0, there can be different forms if 0-0 is extracted from a different sub-component. For example, for the same 0-0 interactions, it is possible that the interaction is extracted from 0-0-0, 0-0-1, 0-0-2,etc.

It is easy to imagine that when the complex grows larger and larger, the length of the list of the possible moves will also increase at a rapid rate. However, most of the moves are impossible due to overlapping or the chain having no space to add a new chain. Therefore, to reduce the number of unlikely moves, only chains with [minimum interaction +1] will be selected for adding new chains. The idea is relatively simple and inspired by how the human brain would add chains to a complex. For example, for a halfway-finished protein, it is easy for humans to think we should add chains on the two ends to assemble the structure (Figure S3, S4). However, if we do not filter for possible moves, the MCTS will try to add a new chain on every chain and see which move is the best. This would increase the computation time largely due to inefficient searching. Although the filtering sounds permissible, it only works well for symmetrical proteins. For asymmetrical protein, the filtering would even make it harder to assemble the protein since the number of interactions does not help us prioritise the searching order. If no node can be expanded, the node with the highest UCB score will be selected and simulation will be performed on that node.

A simulation is performed on the structure with the added chain for the simulation step. During the simulation step, a new chain is randomly added to the structure, and the potential moves list is generated the same way in expansions. The final complex from the simulation is then scored using the pDockQ equation (Bryant, Pozzati, & Elofsson, 2022):

$$log10\ (no.\ of\ interface\ contacts) \cdot\ average\ interface\ plDDT$$

For the back-propagation step, the score from simulation will be back-propagated from the current node to all its parent nodes until the root node.

For the selection step, the best moves is selected using the upper confidence bound (UCB) equation:

$$UCB\ =\ Vi\ +\ \sqrt{\frac{ln\,N}{ni}}$$

where Vi is the average complex score of all nodes below node i, N is the number of times the parent node has been visited, and ni is the number of times the node being scored has been visited.

The MCTS procedure is continued until it reaches the maximum number of moves or no more non-overlapping chains can be added to the complex. At this point, the procedure is terminated.

The MCTS procedure is outlined as a pseudo-code below:

```
for move in moves: # number of protein chains can be added
      # 1. Selection
```

```
current node = best leaf node(current node) while current
node not fully expanded :
# 2. Expansion
current node.expand()
for step in steps : # simulation steps
      # 3. Simulation
      score = simulate(current node)
      # 4. Back-propagation back prop(score)
```

1.4 Evaluation

In the current study, the DockQ program is deemed impractical for evaluating large protein complexes, given its lengthy computation time (ranging from minutes to hours for a single complex) when comparing all interfaces. As an alternative, the MMalign program is employed to assess an entire complex, following the prior scoring of dimeric complexes with FoldDock. MMalign offers optimal structural alignment between the native structures and models, and produces a normalized TM-score between zero and one, with a value of one indicating a perfect match.

One advantage of using MMalign is its ability to score models of varying sizes, which is crucial in the context of this study as predictions are based on full-length protein sequences and incomplete assemblies, with loops and other disordered regions missing from PDB structures. Consequently, the scores for most proteins can never reach 1 and depend on the similarity between the SEQRES sequence and that present in the PDB structure. This study represents a more realistic approach by employing the actual sequences in the evaluation process.

1.5 Computational time

The number of constituent subcomponents primarily influences the computational time required for predicting the structure of a molecular complex. In the case of subcomponent prediction using Alphafold Multimer (AFM), the initial step involves the generation of Multiple Sequence Alignments (MSAs), which takes approximately 1-2 hours on the NVIDIA A100 Tensor Core. This duration is contingent upon the MSA's size and the sequences' lengths. After the MSA generation, the AFM folding process consumes approximately 3-4 hours on NVIDIA A100 Tensor Core GPUs for each protein subcomponent. This timeframe is mainly dependent on the dimensions of the complexes and MSAs. Consequently, the overall prediction time per subcomponent using AFM falls within 5–6 hours.
Following structural analysis, where highly similar subcomponents are removed, the average number of trimeric/tetrameric subcomponents is approximately 8 for the 175

molecular complexes. This results in an average total prediction time of 40-48 hours for trimeric/tetrameric subcomponents within each complex using AFM. The assembly time remains relatively consistent, regardless of the method used for subcomponent prediction, averaging 3-5 hours per complex when considering all trimeric/tetrameric subcomponents. This duration scales exponentially with the increasing number of possible paths. For structures with a straightforward searching path, such as cyclic homomers, the entire assembly process is completed in less than 30 minutes.
The search depth, a parameter defined by the user in the computational pipeline, impacts the assembly time. A shallower search depth reduces the assembly time but increases the likelihood of inaccurate predictions. The default search depth for this study is set at 20, indicating that a maximum of 20 simulation steps are performed for each Monte Carlo Tree Search (MCTS) cycle. The search is prioritised based on the number of interfaces with other molecular chains, making MCTS more efficient than random searching. As a result, the assembly time is negligible compared to the time required to predict the structure of subcomponents and relies solely on Central Processing Unit (CPU) resources.

1.6 Dataset

The same dataset used in MoLPC is used in this project and as the bench-marking dataset for comparison between MoLPC, AlphaFold-Multimer and our new pipeline. All complexes with 10-30 chains from the PDB are obtained on 2022-01-10. First, we selected all complexes not containing nucleic acids with ¡= 3 ˚A resolution and experimental method X-ray crystallography or Electron Microscopy (1216). From these complexes, we require that all chains originate from the same organism (1027). We cluster all sequences from the complexes on 20% sequence identity using MMseqs2 (version edb8223d1ea07385ffe63d4f103af0eb12b2058e) using this command:

```
MMseqs2 easy-cluster fastafile outname /tmp --min-seq-id 0.2
-c 0.8 --cov-mode 1
```

We utilised a clustering approach to ensure that the sub-components of each complex do not overlap with those of any other complex. Specifically, we retained the complexes with the most significant number of clusters, leading to the removal of sub-components from larger clusters (totalling 265). For instance, if the sequences from complex 1 mapped to clusters A, B, and C, while those of complex 2 mapped to clusters A, B, C, and D, then complex 2 was retained, and complex 1 was excluded. Following clustering, we eliminated any complex containing a chain shorter than 50 residues (193 complexes) to remove protein-peptide interactions.

Subsequently, we downloaded the first biological assembly of each complex, ensuring that the reported stoichiometry was accurate and that the PDB files did not contain discontinuous chains, resulting in a total of 175 complexes. The number of chains in the complexes was distributed, with most containing 10-12 chains, averaging 22 interactions and 70 contacts between each pair of interacting chains. The definitions of symmetry were obtained from the PDB annotation, specifically, global symmetry.

For detailed instructions for re-constructing the dataset, please refer to the original MoLPC GitHub and paper (Bryant, Pozzati, Zhu, et al., 2022).

References

Bryant, P., Pozzati, G., & Elofsson, A. (2022). Improved prediction of protein-protein interactions using AlphaFold2. *Nature Communications*, *13*(1), Article 1. https://doi.org/10.1038/s41467-022-28865-w

Bryant, P., Pozzati, G., Zhu, W., Shenoy, A., Kundrotas, P., & Elofsson, A. (2022). Predicting the structure of large protein complexes using AlphaFold and Monte Carlo tree search. *Nature Communications*, *13*(1), Article 1. https://doi.org/10.1038/s41467-022-33729-4

Burke, D. F., Bryant, P., Barrio-Hernandez, I., Memon, D., Pozzati, G., Shenoy, A., Zhu, W., Dunham, A. S., Albanese, P., Keller, A., Scheltema, R. A., Bruce, J. E., Leitner, A., Kundrotas, P., Beltrao, P., & Elofsson, A. (2023). Towards a structurally resolved human protein interaction network. *Nature Structural & Molecular Biology*, *30*(2), Article 2. https://doi.org/10.1038/s41594-022-00910-8

Labbé, Y., Zagoruyko, S., Kalevatykh, I., Laptev, I., Carpentier, J., Aubry, M., & Sivic, J. (2020). *Monte-Carlo Tree Search for Efficient Visually Guided Rearrangement Planning* (arXiv:1904.10348). arXiv. https://doi.org/10.48550/arXiv.1904.10348

Metropolis, N., & Ulam, S. (1949). The Monte Carlo Method. *Journal of the American Statistical Association*, *44*(247), 335–341. https://doi.org/10.1080/01621459.1949.10483310

Mukherjee, S., & Zhang, Y. (2009). MM-align: A quick algorithm for aligning multiple-chain protein complex structures using iterative dynamic programming. *Nucleic Acids Research*, *37*(11), e83. https://doi.org/10.1093/nar/gkp318

Sadeghnejad-Barkousaraie, A., Bohara, G., Jiang, S., & Nguyen, D. (2020). *A reinforcement learning application of guided Monte Carlo Tree Search algorithm for beam orientation selection in radiation therapy* (arXiv:2004.06244). arXiv. https://doi.org/10.48550/arXiv.2004.06244

Silver, D., Hubert, T., Schrittwieser, J., Antonoglou, I., Lai, M., Guez, A., Lanctot, M., Sifre, L., Kumaran, D., Graepel, T., Lillicrap, T., Simonyan, K., & Hassabis, D. (2017). *Mastering Chess and Shogi by Self-Play with a General Reinforcement Learning Algorithm* (arXiv:1712.01815). arXiv. https://doi.org/10.48550/arXiv.1712.01815

Van den Broeck, G., Driessens, K., & Ramon, J. (2009). Monte-Carlo Tree Search in Poker Using Expected Reward Distributions. In Z.-H. Zhou & T. Washio (Eds.), *Advances in Machine Learning* (pp. 367–381). Springer. https://doi.org/10.1007/978-3-642-05224-8_28

Wallner, B. (2023). *AFsample: Improving Multimer Prediction with AlphaFold using Aggressive Sampling* (p. 2022.12.20.521205). bioRxiv. https://doi.org/10.1101/2022.12.20.521205
